# Hemostasis functions are associated with hemorrhagic transformation in non-atrial fibrillation patients: a case-control study

**DOI:** 10.1186/s12883-021-02065-3

**Published:** 2021-01-26

**Authors:** Hao-Ran Cheng, Yun-Bin Chen, Ya-Ying Zeng, Yi-Ting Ruan, Cheng-Xiang Yuan, Qian-Qian Cheng, Hui-Jun Chen, Xiao-Qian Luan, Gui-Qian Huang, Jin-Cai He

**Affiliations:** 1 Department of Neurology, The First Affiliated Hospital of Wenzhou Medical University, Wenzhou, 325000 Zhejiang China; 2 School of Mental Health, Wenzhou Medical University, Wenzhou, 325000 Zhejiang China

**Keywords:** Hemorrhagic transformation, Hemostasis function, Atrial fibrillation, Acute ischemic stroke

## Abstract

**Background:**

Hemorrhagic transformation (HT) is a serious neurological complication of acute ischemic stroke (AIS) after revascularization. The majority of AIS patients do not have atrial fibrillation (AF) which could also develop into HT. In this study, we aimed to explore whether hemostasis parameters are risk factors of HT in non-AF patients.

**Methods:**

We consecutively enrolled 285 AIS patients with HT. Meanwhile, age- and sex-matched 285 AIS patients without HT were included. The diagnosis of HT was determined by brain CT or MRI during hospitalization. All patients were divided into two subgroups based on the presence of AF and explore the differences between the two subgroups. Blood samples were obtained within 24 h of admission, and all patients were evenly classified into three tertiles according to platelet counts (PLT) levels.

**Results:**

In this study, we found the first PLT tertile (OR = 3.509, 95%CI = 1.268–9.711, *P* = 0.016) was independently associated with HT in non-AF patients, taking the third tertile as a reference. Meanwhile, we also found mean platelet volume (MPV) (OR = 0.605, 95%CI = 0.455–0.805, *P* = 0.001) and fibrinogen (FIB) (OR = 1.928, 95%CI = 1.346–2.760, *P* < 0.001) were significantly associated with HT in non-AF patients. But in AF patients, hemostasis parameters showed no significant difference. Meanwhile, we found the MPV (OR = 1.314, 95%CI = 1.032–1.675, *P* = 0.027) and FIB (OR = 1.298, 95%CI = 1.047–1.610, *P* = 0.018) were significantly associated with long-term outcomes in non-AF HT patients.

**Conclusions:**

Low PLT, low MPV, and high FIB levels were independently associated with HT in non-AF patients. Additionally, MPV and FIB levels were significantly associated with unfavorable long-term outcomes in non-AF HT patients. Our study showed that hemostasis functions at admission may be beneficial for clinicians to recognize patients with a high risk of HT at an early stage and improve unfavorable long-term outcomes in non-AF patients.

**Supplementary Information:**

The online version contains supplementary material available at 10.1186/s12883-021-02065-3.

## Background

Hemorrhagic transformation (HT), one of the most common neurological complications after acute ischemic stroke (AIS), is associated with early mortality and poor outcomes after stroke [1–6]. Currently, many risk factors related to HT have been identified including old age, the severity of a stroke, dyslipidemia, hyperglycemia, hemoglobin A1c, atrial fibrillation (AF), and thrombolytic therapy [7–9].

It has been reported that AF and some medication used for its treatment such as Warfarin could cause HT [10–13]. In 2019, Jiao et al. found that AF independently correlated with HT and was a risk factor of HT [8]. In a prospective trial of 101 AIS patients, Tu et al. found that AF patients had significant hypoperfusion that may damage vascular integrity leading to a more frequent HT [12]. Meanwhile, Altavilla et al. found that patients with AF who received low-molecular-weight heparin have a higher risk of HT than non-bridged patients [14]. Besides, an animal experiment found that the incidence of HT in rats treated with Warfarin was increased [13].

However, HT has been underexplored in non-AF patients; the majority of AIS patients are non-AF patients [15, 16] and they could also develop HT. Meanwhile, AF and medication for AF would influence hemostasis functions [17, 18]. Thus, the risk factors of non-AF patients to develop HT after AIS need to be further explored, especially hemostasis functions.

In this research, we mainly explored the association between hemostasis functions and HT in patients with AF and without AF and aimed to identify hemostatic parameters as risk factors in non-AF HT patients.

## Methods

### Subjects

This was a retrospective study of patients with and without hemorrhagic transformation after stroke. The study protocol obtained the approval of the Ethics Committee of the First Affiliated Hospital of Wenzhou Medical University. We didn’t have informed consent for it was a retrospective study and the patient profile was anonymous. The cohort was made up of the First Affiliated Hospital of Wenzhou Medical University’s clinical database of HT; the same amounts of stroke patients without HT from our stroke center were matched by age and sex using the same inclusion and exclusion criteria. All patients who were diagnosed with HT in the First Affiliated Hospital of Wenzhou Medical University were included in this study from December 2013 to December 2015. The inclusion criteria were as follows: (1) age between 18 and 99 years; (2) patient was included within 7 days of stroke; (3) the diagnosis of stroke was confirmed by computerized tomography (CT) or magnetic resonance imaging (MRI) at the time of admission. The exclusion criteria were as follows: (1) hemorrhagic stroke or transient ischemic attack (TIA); (2) patient received intravenous thrombolytic therapy; (3) patients with any severe liver or kidney dysfunction; (4) patients failed to receive a repeat CT/MRI scan; (5) patients’ medical record was incomplete.

### Diagnosis of HT

All patients received a brain CT scan or MRI including diffusion-weighted imaging (DWI) and T2-weighted gradient-echo within 24 h after stroke onset. HT was diagnosed in a subsequent CT/MRI performed 7 ± 2 days after stroke onset or whenever a worsening clinical condition. Two neurologists evaluated the CT/MRI scans independently and diagnosed HT, who were blinded to the results of clinical and laboratory measurements. In this study, HT was categorized radiologically as follows according to the recommendations of the European Cooperative Acute Stroke Study [19, 20]. Hemorrhagic infarct types 1 and 2 were defined as punctate petechiae along the margins of the infarction (HI-1); and more confluent petechiae within the infarcted area, but no space-occupying effect (HI-2). Parenchymal hematoma types 1 and 2 and were defined as hematoma with slight space-occupying effect (≤ 30% infarcted area) (PH-1); and hematoma with significant space-occupying effect (> 30% infarcted area) or intraparenchymal hemorrhage outside the infarcted area (PH-2).

### Data collection

Patients’ demographic data and personal hobbies included gender, age, current smoking, and drinking. Past medical history included the previous history of stroke, hypertension, diabetes mellitus, coronary artery disease (CAD), atrial fibrillation, and hyperlipidemia. Meanwhile, the National Institutes of Health Stroke Scale (NIHSS) was used to evaluate the severity of stroke within 24 h after admission. All patients identified the stroke subtypes according to the TOAST criteria [21]. Blood samples and blood pressure were obtained within 24 h of hospital admission and sent blood samples for tests as soon as possible. Laboratory tests included leukocyte counts, erythrocyte counts, platelet counts (PLT), mean platelet volume (MPV), prothrombin time (PT), international normalized ratio (INR), and fibrinogen (FIB). Also, drug applications in the hospital including anticoagulant, antiplatelet, and lipid-lowering drugs were collected and documented. All patients were evenly classified into three tertiles according to the PLT levels (tertile 1, < 175; tertile 2, 175–222; and tertile 3, > 222).

In December 2019, HT patients were followed up by telephone to collect their up-to-date functional conditions. We used the modified Rankin Scale (mRS) to assess long-term functional outcomes, and an unfavorable functional outcome was defined as an mRS score of ≥2 [22–25]. The primary outcome was defined as mRS.

### Statistical analyses

Statistical descriptions of continuous variables were mean ± SD or medians (quartiles), while categorical variables were percentages. The comparison of continuous variables used the Student’s t-test or Mann-Whitney test, while categorical variables used the Chi-square test or Fisher’s exact test. The comparison of the differences between three PLT tertiles in continuous variables used the Kruskal-Wallis test or one-way analysis of variance (ANOVA), while categorical variables used the Pearson’s chi-square or Fisher’s exact tests. The same way used in the comparison of the differences between the other three groups. Multiple logistic regression analysis was performed to identify significant independent related factors of HT and adjust potential confounding factors. Cox proportional hazards model was performed to identify the significant independent related factors of unfavorable long-term outcomes in non-AF HT patients and adjust potential confounding factors. All statistical analyses used IBM SPSS Statistics for Windows, Version: 19.0.0 (Chicago, IL). Two-tailed *P*-values < 0.05 were considered statistically significant.

## Results

### Baseline characteristics of patients stratified by HT

In this study, 285 AIS patients with HT and age- and sex-matched 285 AIS patients without HT were included. The baseline demographic, clinical, and laboratory characteristics of the study patients were presented in Table 1. The mean age of the enrolled patients was 68.9 ± 12.4 years. 388 (68.1%) patients were male and 182 (31.9%) were female. Also, 137 (24.0%) patients had a history of AF. As for the etiological factors of stroke, among all the enrolled patients, 405 (75.6%) patients were because of atherosclerosis, 109 (20.3%) patients were because of cardioembolism, 4 (0.7%) patients were because of small vessel occlusion and 18 (3.3%) patients were because of other causes. Separately, the proportion of cardioembolism among the HT patients was more than the non-HT patients (*P* < 0.001, Table 1). As shown in Table 1, patients with HT had lower baseline systolic blood pressure (SBP), PLT, and MPV; higher leukocyte counts, PT, INR, and FIB compared with patients without HT. They also had higher NIHSS scores at admission. AF was more frequently found in patients with HT and the proportion of AF among HT and non-HT patients was 108 (37.9%) and 29 (10.2%) respectively. As for the anticoagulant and antiplatelet therapy, non-HT patients had a higher proportion of antiplatelet therapy (*P* < 0.001, Table 1) than HT patients, and HT patients had a higher proportion of anticoagulant therapy (*P* < 0.001, Table 1) than non-HT patients. Separately, non-HT patients also had a higher proportion of antiplatelet therapy than HT patients among the AF group and non-AF group (*P* = 0.044 and *P* < 0.001, respectively; Supplementary Table 1. There was no significant difference in anticoagulant therapy among the AF group. Non-HT patients had a higher proportion of double antiplatelet therapy than HT patients (*P* = 0.025, Table 1). In non-AF patients, 237 (92.6%) non-HT patients and 115 (65.0%) HT patients received antiplatelet therapy; 56 (21.9%) non-HT patients and 25 (14.1%) HT patients received double antiplatelet therapy (Supplementary Table 1). And the antiplatelet agents included Aspirin and Clopidogrel. Besides, patients with HT were more likely to smoke and drink.

**Table 1:** Baseline characteristics of AIS patients without HT and with HT

Variables	Total (*n*=570)	Non-HT (*n*=285)	HT (*n*=285)	*P*-value
Demographic characteristics				
Age (years)	68.9 ± 12.4	68.9 ± 12.3	68.9 ± 12.6	0.970
Male, n (%)	388 (68.1%)	191 (67.0%)	197 (69.1%)	0.590
Baseline SBP (mmHg)	153.3 ± 23.1	158.0 ± 23.0	148.6 ± 22.3	< 0.001
Baseline DBP (mmHg)	82.3 ± 13.8	82.1 ± 13.3	82.5 ± 14.3	0.732
NIHSS on admission, median (IQR)	5.0 (2.0–10.0)	3.0 (1.0–5.0)	9.0 (5.0–13.0)	< 0.001
CTA, n (%)	64 (11.2%)	30 (10.5%)	34 (11.9%)	0.616
Vascular risk factors, n (%)				
Current smoking	210 (37.1%)	118 (41.5%)	92 (32.6%)	0.028
Current drinking	228 (40.4%)	141 (49.8%)	87 (30.9%)	< 0.001
Previous Stroke	72 (12.6%)	31 (10.9%)	41 (14.4%)	0.207
Hypertension	376 (66.0%)	197 (69.1%)	179 (62.8%)	0.112
Diabetes	149 (26.1%)	80 (28.1%)	69 (24.2%)	0.294
CAD	47 (8.3%)	15 (5.3%)	32 (11.3%)	0.010
Dyslipidemia	37 (6.5%)	17 (6.0%)	20 (7.0%)	0.610
AF	137 (24.0%)	29 (10.2%)	108 (37.9%)	< 0.001
Hematological variables				
Leukocyte counts (×10^9^/L)	7.7 ± 2.8	6.8 ± 1.9	8.5 ± 3.3	< 0.001
Erythrocyte counts (×10^9^/L)	4.4 ± 0.6	4.4 ± 0.6	4.4 ± 0.6	0.836
PLT (× 10^9^/L), median (IQR)	197.0 (162.0–235.0)	205.0 (175.0–238.5)	187.0 (148.5–231.0)	< 0.001
MPV (fl)	11.0 ± 1.4	11.2 ± 1.2	10.8 ± 1.4	0.011
PT (s)	13.7 ± 1.0	13.5 ± 1.0	13.9 ± 1.0	< 0.001
INR	1.1 ± 0.1	1.0 ± 0.1	1.1 ± 0.1	< 0.001
FIB (g/L)	3.8 ± 1.6	3.5 ± 1.0	4.1 ± 2.0	< 0.001
Stroke etiology, n (%)				< 0.001
Atherosclerosis	405 (75.6%)	214 (84.9%)	191 (67.3%)	
Cardioembolism	109 (20.3%)	20 (7.9%)	89 (31.3%)	
Small vessel occlusion	4 (0.7%)	3 (1.2%)	1 (0.4%)	
Other causes	18 (3.3%)	15 (6.0%)	3 (1.1%)	
Treatment, n (%)				
Anticoagulant therapy	114 (20%)	28 (9.8%)	86 (30.2%)	< 0.001
Antiplatelet therapy	419 (73.5%)	256 (89.8%)	163 (57.2%)	< 0.001
Aspirin	201 (35.3%)	120 (42.1%)	81 (28.4%)	0.001
Clopidogrel	122 (21.4%)	78 (27.4%)	44 (15.4%)	< 0.001
Double antiplatelet therapy	96 (16.8%)	58 (20.4%)	38 (13.3%)	0.025

Abbreviations: HT, hemorrhagic transformation; SBP, systolic blood pressure; DBP, diastolic blood pressure; NIHSS, National Institute of Health Stroke Scale; CTA, computered tomograhy angiography; CAD, coronary artery disease; AF, atrial fibrillation; PLT, platelet counts; MPV, mean platelet volume; PT, prothrombin time; INR, International Normalized Ratio; FIB, fibrinogen

According to the radiological features, HT patients were classified to 61 (21.4%) HI-1, 86 (30.2%) HI-2, 69 (24.2%) PH-1 and 69 (24.2%) PH-2. In the Supplemental Table 2, PH patients had lower PLT, lower FIB and higher MPV than HI patients.

### Baseline characteristics of AIS patients according to PLT tertiles

Demographic and laboratory variables according to PLT tertiles were presented in Table 2. The incidence of HT was significantly higher in the first PLT tertile than the second and third MLR tertiles (63.7% versus 42.8 and 43.2%, respectively; *P* < 0.001). As shown in Table 2, patients with lower PLT levels were more likely to be drinkers and CAD patients; had lower leukocyte counts and FIB levels, and had higher MPV, PT, and INR levels; and were more likely to receive anticoagulant therapy, less likely to receive antiplatelet therapy.

**Table 2:** Baseline characteristics of AIS patients according to PLT tertiles

Variables	PLT tertiles			
	Tertile1 (*n*=193)	Tertile2 (*n*=187)	Tertile3 (*n*=190)	*P*-value
PLT (×109/L)	< 175	175–222	> 222	
HT, n (%)	123 (63.7%)	80 (42.8%)	82 (43.2%)	< 0.001
**Demographic characteristics**				
Age (years)	70.2 ± 11.6	68.9 ± 12.7	67.5 ± 12.8	0.970
Male, n (%)	147 (76.2%)	130 (69.5%)	111 (58.4%)	0.590
Baseline SBP (mmHg)	150.0 ± 22.2	157.8 ± 21.8	152.3 ± 24.5	< 0.001
Baseline DBP (mmHg)	81.6 ± 14.0	83.2 ± 14.2	82.2 ± 13.2	0.732
NIHSS on admission, median (IQR)	5.0 (3.0–11.0)	4.5 (2.0–9.0)	5.0 (2.0–9.0)	0.028
CTA, n (%)	16 (8.3%)	26 (13.9%)	22 (11.6%)	0.218
**Vascular risk factors, n (%)**				
Current smoking	64 (33.2%)	64 (34.2%)	82 (43.2%)	0.028
Current drinking	80 (41.5%)	74 (39.6%)	74 (38.9%)	< 0.001
Previous Stroke	26 (13.5%)	20 (10.7%)	26 (13.7%)	0.207
Hypertension	115 (59.6%)	132 (70.6%)	129 (67.9%)	0.112
Diabetes	45 (23.3%)	56 (29.9%)	48 (25.3%)	0.294
CAD	23 (11.9%)	14 (7.5%)	10 (5.3%)	0.010
Dyslipidemia	12 (6.2%)	13 (7.0%)	12 (6.3%)	0.610
AF	63 (32.6%)	41 (21.9%)	33 (17.4%)	< 0.001
**Hematological** variables				
Leukocyte counts (×10^9^/L)	7.4 ± 2.8	7.8 ± 3.0	7.8 ± 2.6	< 0.001
Erythrocyte counts (×10^9^/L)	4.4 ± 0.6	4.5 ± 0.6	4.4 ± 0.5	0.836
MPV (fl)	11.3 ± 1.5	10.9 ± 1.4	10.6 ± 1.2	0.007
PT (s)	14.0 ± 1.1	13.6 ± 1.0	13.5 ± 1.0	< 0.001
INR	1.1 ± 0.1	1.0 ± 0.1	1.0 ± 0.1	< 0.001
FIB (g/L)	3.7 ± 1.2	3.7 ± 1.1	4.0 ± 2.3	< 0.001
**Stroke etiology, n (%)**				< 0.001
Atherosclerosis	126 (68.4%)	137 (73.3%)	142 (74.7%)	
Cardioembolism	51 (26.4%)	29 (15.5%)	29 (15.3%)	
Small vessel occlusion	2 (1.0%)	1 (0.5%)	1 (0.5%)	
Other causes	6 (3.1%)	8 (4.2%)	4 (2.1%)	
**Treatment, n (%)**				
Anticoagulant therapy	61 (31.6%)	26 (13.9%)	27 (14.2%)	< 0.001
Antiplatelet therapy	122 (63.2%)	150 (80.2%)	142 (74.7%)	< 0.001

Abbreviations: PLT, platelet counts; HT, hemorrhagic transformation; SBP, systolic blood pressure; DBP, diastolic blood pressure; NIHSS, National Institute of Health Stroke Scale; CTA, computered tomograhy angiography; CAD, coronary artery disease; AF, atrial fibrillation; MPV, mean platelet volume; PT, prothrombin time; INR, International Normalized Ratio; FIB, fibrinogen

### Characteristics of patients with HT in subcategorized groups of AF

The incidence of HT was higher in the AF subgroup than in non-AF (78.8% vs. 40.9%, *P*< 0.001). In the subgroup of non-AF patients, the significant parameters between HT and non-HT (Supplemental Table 3) were generally consistent with the parameters in Table 1. But in AF patients, patients with HT had higher levels of leukocyte counts, lower SBP; higher NIHSS scores at admission; and more smokers and drinkers. In AF patients, there were no significant differences in hemostasis parameters among HT and non-HT patients.

### Association between hemostasis parameters and HT

The occurrence of HT was used as a dependent variable and the third PLT tertile was used as a reference in the multivariate regression in all patients. After adjusting for confounding and risk factors, multivariate regression analysis showed that the first PLT tertile (OR =3.517, 95%CI = 1.526–8.106, *P* = 0.003; Table 3) was independently associated with HT in all patients. Meanwhile, MPV (OR =0.698, 95%CI = 0.557–0.875, *P* = 0.002; Table 3) and FIB (OR = 1.613, 95%CI = 1.199–2.169, P = 0.002; Table 3) were also significantly associated with HT in all patients.

**Table 3:** Multivariate logistic regression analysis of predictive factors for HT in all patients

	Model 1		Model 2		Model 3	
	adjust OR(95%CI)	*P*-value	adjust OR(95%CI)	*P*-value	adjust OR(95%CI)	*P*-value
PLT						
T1	2.002 (1.160–3.454)	0.013	3.887 (1.762–8.575)	0.001	3.517 (1.526–8.106)	0.003
T2	1.055 (0.617–1.803)	0.845	1.425 (0.710–2.861)	0.319	1.525 (0.738–3.149)	0.254
T3	Ref		Ref		Ref	
MPV	–	–	0.714 (0.573–0.890)	0.003	0.698 (0.557–0.875)	0.002
PT	–	–	3.624 (1.015–12.939)	0.047	3.599 (0.941–13.767)	0.061
INR	–	–	0.904 (0.796–1.027)	0.121	0.904 (0.791–1.035)	0.143
FIB	–	–	1.570 (1.191–2.069)	0.001	1.613 (1.199–2.169)	0.002

**Notes:** Model 1: adjusted sex, age, smoking, drinking, CAD, AF, baseline SBP, NIHSS on admission; Model 2: adjusted for covariates from Model 1 and further adjusted for MPV, PT, INR, FIB and leukocyte counts; Model 3: adjusted for covariates from Model 2 and further adjusted for anticoagulant therapy and antiplatelet therapy

**Abbreviations:** CI, confidence interval; OR, odds ratio; HT, hemorrhagic transformation; PLT, platelet counts; MPV, mean platelet volume; PT, prothrombin time; INR, International Normalized Ratio; FIB, fibrinogen

Considering the potential relationship between AF and hemostasis function, we divided all patients into AF and non-AF subgroups. In non-AF subgroup, we found the first PLT tertile (OR = 3.509, 95%CI = 1.268–9.711, *P* = 0.016; Table 4), MPV (OR = 0.605, 95%CI = 0.455–0.805, *P* = 0.001; Table 4) and FIB (OR = 1.928, 95%CI = 1.346–2.760, *P* < 0.001; Table 4) were significantly associated with HT after adjusting for confounding factors. In the AF subgroup, hemostasis parameters showed no significant association with HT.

**Table 4:** Multivariate logistic regression analysis of predictive factors for HT after subcategorized by AF

	Non-AF						AF					
	Model 1		Model 2		Model 3		Model 1		Model 2		Model 3	
	adjust OR (95%CI)	*P*-value	adjust OR (95%CI)	*P*-value	adjust OR (95%CI)	*P*-value	adjust OR (95%CI)	*P*-value	adjust OR (95%CI)	*P*-value	adjust OR (95%CI)	*P*-value
PLT												
T1	2.236 (1.220–4.095)	0.009	4.908 (1.930–12.481)	0.001	3.509 (1.268–9.711)	0.016	1.894 (0.467–7.685)	0.371	3.084 (0.448–21.206)	0.252	4.822 (0.561–41.468)	0.152
T2	0.920 (0.508–1.666)	0.784	1.383 (0.626–3.058)	0.423	1.344 (0.575–3.143)	0.496	1.985 (0.440–8.947)	0.372	1.885 (0.289–12.320)	0.508	1.873 (0.262–13.378)	0.532
T3	Ref		Ref		Ref		Ref		Ref		Ref	
MPV	–	–	0.620 (0.479–0.804)	< 0.001	0.605 (0.455–0.805)	0.001	–	–	1.118 (0.635–1.968)	0.700	1.134 (0.648–1.985)	0.659
PT	–	–	3.774 (0.886–16.074)	0.072	3.500 (0.721–16.997)	0.120	–	–	2.865 (0.093–88.653)	0.548	3.861 (0.102–146.521)	0.466
INR	–	–	0.901 (0.778–1.042)	0.159	0.908 (0.774–1.065)	0.237	–	–	0.927 (0.660–1.304)	0.665	0.902 (0.627–1.298)	0.580
FIB	–	–	1.838 (1.324–2.551)	< 0.001	1.928 (1.346–2.760)	< 0.001	–	–	1.023 (0.726–1.441)	0.897	1.061 (0.735–1.533)	0.752

**Notes:** Model 1: adjusted sex, age, smoking, drinking, CAD, AF, baseline SBP, NIHSS on admission; Model 2: adjusted for covariates from Model 1 and further adjusted for MPV, PT, INR, FIB and leukocyte counts; Model 3: adjusted for covariates from Model 2 and further adjusted for anticoagulant therapy and antiplatelet therapy

**Abbreviations:** CI, confidence interval; OR, odds ratio; HT, hemorrhagic transformation; AF, atrial fibrillation; PLT, platelet counts; MPV, mean platelet volume; PT, prothrombin time; INR, International Normalized Ratio; FIB, fibrinogen

After adjusting for confounding factors, we found MPV (OR = 0.744, 95%CI = 0.594–0.931, *P* = 0.010; Supplemental Table 3) were significantly associated with HI. Meanwhile, the first PLT tertile (OR = 3.719, 95%CI = 1.551–8.916, *P* = 0.003; Supplemental Table 3) were significantly associated with PH after adjusting for confounding factors.

### Results of long-term functional outcomes after HT

Of 285 HT patients, 143 (50.2%) were available for follow-up in December 2019 through phone calls and the median follow-up time was 4.75 years (IQR 2.82–6.14). Among 143 follow-up patients, 85 were non-AF patients. In the long term, 58 (68.2%) of the non-AF HT patients had unfavorable functional outcomes (mRS≥2). After adjusting for confounding factors, Cox proportional hazards regression analysis showed that MPV (OR = 1.314, 95%CI = 1.032–1.675, *P* = 0.027; Table 5) and FIB (OR = 1.298, 95%CI = 1.047–1.610, *P* = 0.018; Table 5) was independently and significantly associated with long-term outcomes in non-AF HT patients.

**Table 5:** Cox proportional hazards model of predictive factors for long-term outcome in non-AF HT patients

	HR	95% CI	*P*-value
Sex	0.618	0.304–1.258	0.184
Age	1.020	0.985–1.057	0.256
NIHSS on admission	1.065	0.989–1.146	0.094
Hypertension	0.810	0.410–1.602	0.545
Diabetes	2.706	1.170–6.254	0.020
CAD	0.977	0.421–2.267	0.956
PLT			
T1	0.674	0.317–1.433	0.306
T2	1.399	0.632–3.098	0.408
T3	Ref		
MPV	1.314	1.032–1.675	0.027
FIB	1.298	1.047–1.610	0.018
Anticoagulant therapy	0.875	0.427–1.794	0.716
Antiplatelet therapy	0.838	0.429–1.636	0.604

**NOTE.** CI, confidence interval; HR, hazard ratio; AF, atrial fibrillation; HT, hemorrhagic transformation; NIHSS, National Institute of Health Stroke Scale; CAD, coronary artery disease; PLT, platelet counts; MPV, mean platelet volume; FIB, fibrinogen

## Discussion

To the best of our knowledge, this is the first study to explore the association of hemostasis parameters and HT in non-AF patients; and to explore the effect of hemostasis parameters on long-term outcomes after HT in non-AF patients. Our present study showed that three hemostasis parameters including low PLT, low MPV, and high FIB were significant and dependent risk factors with HT in non-AF patients, instead of AF patients. Furthermore, we found that MPV and FIB levels were independently and significantly associated with unfavorable long-term functional outcomes in non-AF HT patients.

The etiological factors of stroke include atherosclerosis, cardioembolism, small vessel occlusion, and other causes. In this study, atherosclerosis was the most common cause of stroke among all the patients, which was consistent with previous studies. Atherosclerosis has been proved to be a major pathophysiological finding associated with a large proportion of ischemic stroke [26]. As for the HT patients, the proportion of cardioembolism was more than the non-HT group, which was also consistent with previous studies. Cardio embolism has been proved to be an independent risk factor for HT [27]. Consistent with many studies, we identified that AF was a reliable risk factor of HT, and the incidence of HT among AF patients was higher than non-AF patients [7, 8, 28, 29]. Considering the complex interaction between AF and HT [30], we did a subgroup analysis of our patients to explore the association between hemostasis functions and HT. Considering the complex interaction between AF and HT, we did a subgroup analysis to explore the association between coagulation functions and HT. We found none of the coagulation parameters was associated with HT among AF patients. However, there were very few studies on this topic, so we made some hypotheses based on our findings. The possible reasons are as follows. First, the proportion of antiplatelet therapy among AF patients was lower than non-AF. Thus, it may have little effect on platelet function and platelet count, which could not predict the prevalence of HT. Second, due to the high proportion and prolonged use of anticoagulants before admission among AF patients, coagulation functions of AF patients may worse than non-AF patients. Indeed, in our study, coagulation functions such as the levels of PT (*P* < 0.001), FIB (*P* = 0.003) and INR (P < 0.001) among AF patients were higher than non-AF. However, for AF patients, there was no difference in the proportion of anticoagulant therapy between HT and non-HT groups. Thus, there was no difference in the coagulation parameters between HT and non-HT groups, which could not predict the prevalence of HT.

However, our study suggested that PLT, MPV, and FIB were associated with HT significantly in non-AF patients. PLT is a key factor in the hemostasis process. According to clinical guidelines from the American Heart Association/American Stroke Association published in 2018, low PLT levels (< 100,000 counts/μl) would increase the risk of HT [31] and do not recommend these patients to receive reperfusion therapies. Several studies also pointed out that PLT was associated with the occurrence of HT [8, 32]. Furthermore, a previous study identified that lower coated-platelets counts increased the likelihood of early HT in patients with non-lacunar ischemic stroke [33]. Platelet-endothelial interactions could maintain the structural integrity of blood vessels when a stroke occurs [34]. The patients with low PLT levels are weak in maintaining the blood vessels’ integrity and are more likely to develop HT after AIS. Salas-Perdomo et al. found in the experiment that using an anti-platelet serum in mice would lead to larger intraparenchymal hematomas after stroke [35], thus stressed the vital hemostatic function of platelets in AIS. This may because those platelets can physically cover the damaged vascular endothelium or release protective factors to protect the endothelial barrier function. These all indicated the importance of PLT in HT.

MPV describes the sizes of PLT, which is a marker of PLT activity and influences bleeding [36, 37]. In our study, MPV among HT patients was lower than non-HT. Besides, logistic regression analysis showed low MPV was a risk factor of HT. Early study found that larger PLT had more granules and would produce more vascular activity as well as pre-clotting factors, which may raise the hemostatic efficiency [37, 38]. A retrospective study suggested that baseline MPV was associated with unfavorable stroke outcomes but its relationship with HT was still uncertain [39].

Previous studies briefly mentioned the relationship between FIB and HT [32, 40, 41]. And we found that higher FIB may be associated with a higher incidence of HT among AIS patients, which is congruent with some reports [40, 41]. However, Wang et al. found that FIB < 1.50 g/L was a risk factor for HT [32]. We hypothesize that high levels of FIB may be associated with HT by participating in the inflammatory process. When HT occurs, endothelial dysfunction of capillaries would further lead to abnormal blood-brain barrier permeability within the infarcted area [42]. The dysfunction of the blood-brain barrier in the infarcted area was proved to be the main cause of HT. Meanwhile, a review proposed that FIB was a ligand of cell surface receptors and this could promote the intercellular adhesion between inflammatory cells and endothelial [43]. In another study, an anti-inflammatory thrombolytic drug called SMTP-7 could decrease the incidence of HT, which suggested the relationship between HT and inflammation [44]. Thus, the relationship between FIB and HT remains unknown and this needs to be further investigated.

In our study, we found that hemostasis functions were associated with unfavorable long-term outcomes in non-AF HT patients. Its exact mechanism remains unknown; however, the mechanism by which hemostasis function is related to unfavorable outcomes in intracerebral hemorrhage (ICH) is unclear. Unfavorable outcomes after hemorrhage were reported to be conducted by the inflammatory effects of intraparenchymal blood [45]. Recently, Krenzlin H et al. found that the activated cerebral thrombin system was related to poor outcomes after ICH in mice. They suspected that the activated cerebral thrombin system may contribute to secondary brain damage [46]. Meanwhile, a previous study suggested that hemostasis function may influence neurovascular injury and neuroprotection [47].

Our study has some limitations. First, this was a single-center and retrospective study and we did not have the regular follow-up records such as 1, 3, 5 years. So it is necessary to conduct multi-center, prospective studies to establish causality and provide detailed long-term prognostic information. Second, our study did not discuss the association between different subtypes of HT (hemorrhagic infarction or parenchymal hematoma) and hemostasis functions. In a further study, we could explore the association between the severity of HT and hemostasis functions. Third, owing to the infarction size was not documented, the associations between infarction size and HT were not described in detail. Fourth, our study did not collect the record of endovascular thrombectomy.

## Conclusions

In summary, we found that three hemostasis parameters including low PLT, low MPV, and high FIB were risk factors among the non-AF patients, while these parameters were not associated with HT in AF patients. Meanwhile, MPV and FIB levels were independently and significantly associated with unfavorable long-term outcomes in non-AF HT patients. Our study demonstrated that the hemostasis functions may be useful hematological markers for clinicians to recognize patients with a high risk of HT at an early stage and improve unfavorable long-term outcomes in non-AF patients.

## Supplementary Information

**Additional file 1:** **Table S1.** Baseline characteristics of AIS patients according to subcategorized group by AF

**Additional file 2:** **Table S2.** Baseline characteristics of AIS patients according to the subcategorized groups of HT

**Additional file 3:** **Table S3.** Multivariate logistic regression analysis of predictive factors for HI and HT in non-AF patients

## Data Availability

The data supporting this study are available from the corresponding author for a reasonable request.

## Abbreviations

HT: Hemorrhagic transformation
AIS: acute ischemic stroke
AF: atrial fibrillation
PLT: platelet counts
MPV: mean platelet volume
FIB: fibrinogen
CI: confidence interval
OR: odds ratio
CT: computerized tomography
MRI: magnetic resonance imaging
TIA: transient ischemic attack
DWI: diffusion-weighted imaging
CAD: coronary artery disease
NIHSS: National Institutes of Health Stroke Scale
PT: prothrombin time
INR: international normalized ratio
mRS: modified Rankin Scale
ICH: intracerebral hemorrhage
