# Supplementary material for: Hemostasis functions are associated with hemorrhagic transformation in non-atrial fibrillation patients: a case-control study
Source: BMC Neurol. 2021 Jan 26;21:36. doi: 10.1186/s12883-021-02065-3 (PMC7836156; doi:10.1186/s12883-021-02065-3)
Supplement: Supplementary file 1 — Additional file 1: Table S1. Baseline characteristics of AIS patients according to subcategorized group by AF [file 12883_2021_2065_MOESM1_ESM.docx]

| Supplemental Table 1. Baseline characteristics of AIS patients according to subcategorized group by AF | | | | | | |
| --- | --- | --- | --- | --- | --- | --- |
| Variables | Non-AF |  |  | AF |  |  |
|  | Non-HT(n=256) | HT(n=177) | P-value | Non-HT(n=29) | HT(n=108) | P-value |
| Demographic characteristics |  |  |  |  |  |  |
| Age (years) | 68.1 ± 12.3 | 67.2 ± 12.8 | 0.442 | 75.6 ± 10.3 | 71.6 ± 11.8 | 0.101 |
| Male, n (%) | 175(68.4%) | 121(68.4%) | <0.001 | 16 (55.2%) | 76 (70.4%) | 0.122 |
| Baseline SBP (mmHg) | 157.9 ± 22.8 | 148.6 ± 22.9 | <0.001 | 158.4 ± 24.8 | 148.7 ± 21.3 | 0.038 |
| Baseline DBP (mmHg) | 82.0 ± 13.3 | 81.9 ± 14.7 | 0.931 | 83.3 ± 12.8 | 83.6 ± 13.7 | 0.925 |
| NIHSS on admission, median (IQR) | 3.0(1.0-5.0) | 9.0(4.0-12.5) | <0.001 | 2.0(0.0-4.0) | 10.5(6.0-14.0) | <0.001 |
| CTA, n (%) | 28 (11.2%) | 27 (15.3%) | 0.248 | 2 (6.9%) | 7 (6.5%) | 1.000 |
| Vascular risk factors, n (%) |  |  |  |  |  |  |
| Current smoking | 103 (40.2%) | 69 (39.4%) | 0.867 | 15 (53.6%) | 23 (21.5%) | <0.001 |
| Current drinking | 126 (49.4%) | 62 (35.4%) | 0.004 | 15 (53.6%) | 25 (23.4%) | 0.002 |
| Previous Stroke | 25 (9.8%) | 27 (15.3%) | 0.084 | 6 (20.7%) | 14 (13.0%) | 0.295 |
| Hypertension | 177 (69.1%) | 113 (63.8%) | 0.249 | 20 (69.0%) | 66 (61.1%) | 0.437 |
| Diabetes | 76 (29.7%) | 37 (20.9%) | 0.041 | 4 (13.8%) | 32 (29.6%) | 0.085 |
| CAD | 11 (4.3%) | 21 (11.9%) | 0.003 | 4 (13.8%) | 11 (10.2%) | 0.581 |
| Dyslipidemia | 16 (6.2%) | 17 (9.6%) | 0.196 | 1 (3.4%) | 3 (2.8%) | 0.849 |
| Hematological variables |  |  |  |  |  |  |
| Leukocyte counts (×10^9^/L) | 6.8 ± 1.8 | 8.0 ± 2.6 | <0.001 | 6.6 ± 2.7 | 9.4 ± 4.0 | <0.001 |
| Erythrocyte counts (×10^9^/L) | 4.4 ± 0.6 | 4.4 ± 0.5 | 0.977 | 4.4 ± 0.7 | 4.4 ± 0.6 | 0.969 |
| PLT (×10^9^/L), median (IQR) | 207.0(176.3-241.0) | 189.0(153.0-241.0) | 0.004 | 196.0(148.0-224.0) | 178.5(137.3-217.5) | 0.398 |
| MPV (fl) | 11.2 ± 1.2 | 10.7 ± 1.4 | 0.003 | 11.2 ± 0.9 | 11.0 ± 1.5 | 0.549 |
| PT (s) | 13.4 ± 0.9 | 13.7 ± 1.0 | 0.001 | 14.3 ± 1.4 | 14.3 ± 1.0 | 0.982 |
| INR | 1.0 ± 0.1 | 1.1 ± 0.1 | 0.022 | 1.1 ± 0.2 | 1.1 ± 0.1 | 0.762 |
| FIB (g/L) | 3.4 ± 1.0 | 4.1 ± 1.3 | <0.001 | 3.8 ± 1.0 | 4.3 ± 2.9 | 0.405 |
| Stroke etiology, n (%) |  |  | <0.001 |  |  | 0.097 |
| Atherosclerosis | 207 (92.0%) | 153 (86.9%) |  | 7 (25.9%) | 38 (35.2%) |  |
| Cardioembolism | 1 (0.4%) | 19 (10.8%) |  | 19 (70.4%) | 70 (64.8%) |  |
| Small vessel occlusion | 3 (1.3%) | 1 (0.6%) |  | 0 (0.0%) | 0 (0.0%) |  |
| Other causes | 14 (6.2%) | 3 (1.7%) |  | 1 (3.7%) | 0 (0.0%) |  |
| Treatment, n (%) |  |  |  |  |  |  |
| Anticoagulant therapy | 12 (4.6%) | 39 (22.0%) | <0.001 | 16 (55.2%) | 47 (43.5%) | 0.264 |
| Antiplatelet therapy | 237 (92.6%) | 115 (65.0%) | <0.001 | 19 (65.5%) | 48 (44.4%) | 0.044 |
| Aspirin | 109 (42.6%) | 59 (33.3%) | 0.043 | 10 (34.5%) | 22 (20.4%) | 0.111 |
| Clopidogrel | 72 (28.1%) | 31 (17.5%) | 0.011 | 7 (24.1%) | 13 (12.0%) | 0.101 |
| Double antiplatelet therapy | 56 (21.9%) | 25 (14.1%) | 0.042 | 2 (6.9%) | 13 (12.0%) | 0.431 |
| NOTE. SBP, systolic blood pressure; DBP, diastolic blood pressure; PLT platelet counts; MPV mean platelet volume; PT prothrombin time; INR International Normalized Ratio; FIB fibrinogen; NIHSS, National Institute of Health Stroke Scale; CTA, computered tomograhy angiography; CAD coronary artery disease; AF atrial fibrillation. | | | | | | |
