# Supplementary material for: Hemostasis functions are associated with hemorrhagic transformation in non-atrial fibrillation patients: a case-control study
Source: BMC Neurol. 2021 Jan 26;21:36. doi: 10.1186/s12883-021-02065-3 (PMC7836156; doi:10.1186/s12883-021-02065-3)
Supplement: Supplementary file 2 — Additional file 2: Table S2. Baseline characteristics of AIS patients according to the subcategorized groups of HT [file 12883_2021_2065_MOESM2_ESM.docx]

| Supplemental Table 2. Baseline characteristics of AIS patients according to the subcategorized groups of HT | | | | |
| --- | --- | --- | --- | --- |
| Variables | Non-HT (n=285) | HI (n=147) | PH (n=138) | P-value |
| Demographic characteristics |  |  |  |  |
| Age (years) | 68.9 ± 12.3 | 69.3 ± 11.8 | 68.3 ± 13.4 | 0.792 |
| Male, n (%) | 191 (67.0%) | 100 (68.0%) | 97 (70.3%) | 0.795 |
| Baseline SBP (mmHg) | 158.0 ± 23.0 | 151.5 ± 21.2 | 145.7 ± 23.1 | <0.001 |
| Baseline DBP (mmHg) | 82.1 ± 13.3 | 83.0 ± 13.6 | 82.0 ± 15.1 | 0.787 |
| NIHSS on admission, median (IQR) | 3.0 (1.0-5.0) | 8.0 (4.0-120) | 11.0 (6.0-14.0) | <0.001 |
| CTA, n (%) | 30 (10.5%) | 18 (12.2%) | 16 (11.7%) | 0.872 |
| Vascular risk factors, n (%) |  |  |  |  |
| Current smoking | 118 (41.5%) | 48 (32.9%) | 44 (32.4%) | 0.089 |
| Current drinking | 141 (49.8%) | 44 (30.1%) | 43 (31.6%) | <0.001 |
| Previous Stroke | 31 (10.9%) | 23 (15.6%) | 18 (13.0%) | 0.363 |
| Hypertension | 197 (69.1%) | 98 (66.7%) | 81 (58.7%) | 0.103 |
| Diabetes | 80 (28.1%) | 33 (22.4%) | 36 (26.1%) | 0.452 |
| CAD | 15 (5.3%) | 16 (11.0%) | 16 (11.6%) | 0.035 |
| Dyslipidemia | 17 (6.0%) | 14 (9.5%) | 6 (4.3%) | 0.183 |
| AF | 29 (10.2%) | 47 (32.0%) | 61(44.2%) | <0.001 |
| Hematological variables |  |  |  |  |
| Leukocyte counts (×10^9^/L) | 6.8 ± 1.9 | 8.1 ± 3.1 | 8.9 ± 3.5 | <0.001 |
| Erythrocyte counts (×10^9^/L) | 4.4 ± 0.6 | 4.5 ± 0.6 | 4.4 ± 0.6 | 0.553 |
| PLT (×10^9^/L), median (IQR) | 205.0(175.0-238.5) | 195.0 (161.0-236.0) | 171.5 (142.5-229.3) | 0.045 |
| MPV (fl) | 11.2 ± 1.2 | 10.8 ± 1.5 | 10.9 ± 1.4 | 0.033 |
| PT (s) | 13.5 ± 1.0 | 13.7 ± 1.0 | 14.1 ± 1.1 | <0.001 |
| INR | 1.0 ± 0.1 | 1.1 ± 0.1 | 1.1 ± 0.1 | <0.001 |
| FIB (g/L) | 3.5 ± 1.0 | 4.1 ± 1.3 | 4.0 ± 1.4 | <0.001 |
| Stroke etiology, n (%) |  |  |  | <0.001 |
| Atherosclerosis | 214 (84.9%) | 115 (78.2%) | 76 (55.5%) |  |
| Cardioembolism | 20 (7.9%) | 30 (20.4%) | 59 (43.1%) |  |
| Small vessel occlusion | 3 (1.2%) | 1 (0.7%) | 0 (0.0%) |  |
| Other causes | 15(6.0%) | 1 (0.7%) | 2 (1.5%) |  |
| Treatment, n (%) |  |  |  |  |
| Anticoagulant therapy | 28 (9.8%) | 37 (25.2%) | 49 (35.5%) | <0.001 |
| Antiplatelet therapy | 256 (89.8%) | 87 (59.2%) | 76 (55.1%) | <0.001 |
| Aspirin | 120 (42.1%) | 44 (29.9%) | 37 (26.8%) | 0.002 |
| Clopidogrel | 78 (27.4%) | 24 (16.3%) | 20 (14.5%) | 0.002 |
| Double antiplatelet therapy | 58 (20.4%) | 19 (12.9%) | 19 (13.8%) | 0.080 |
| Abbreviations: HT, hemorrhagic transformation; HI, hemorrhagic infarct; PH, parenchymal hematoma; SBP, systolic blood pressure; DBP, diastolic blood pressure; NIHSS, National Institute of Health Stroke Scale; CTA, computered tomograhy angiography; CAD, coronary artery disease; AF, atrial fibrillation; PLT, platelet counts; MPV, mean platelet volume; PT, prothrombin time; INR, International Normalized Ratio; FIB, fibrinogen. | | | | |
