# Supplementary material for: Hemostasis functions are associated with hemorrhagic transformation in non-atrial fibrillation patients: a case-control study
Source: BMC Neurol. 2021 Jan 26;21:36. doi: 10.1186/s12883-021-02065-3 (PMC7836156; doi:10.1186/s12883-021-02065-3)
Supplement: Supplementary file 3 — Additional file 3: Table S3. Multivariate logistic regression analysis of predictive factors for HI and HT in non-AF patients [file 12883_2021_2065_MOESM3_ESM.docx]

| Supplemental Table 3. Multivariate logistic regression analysis of predictive factors for HI and HT in non-AF patients | | | | |
| --- | --- | --- | --- | --- |
|  | HI |  | PH |  |
|  | adjust OR (95%CI) | P-value | adjust OR (95%CI) | P-value |
| PLT |  |  |  |  |
| T1 | 0.778 (0.361-1.676) | 0.521 | 3.719 (1.551-8.916) | 0.003 |
| T2 | 1.056 (0.540-2.064) | 0.874 | 1.243 (0.510-3.031) | 0.632 |
| T3 | Ref |  | Ref |  |
| MPV | 0.744 (0.594-0.931) | 0.010 | 0.917 (0.728-1.155) | 0.461 |
| PT | 2.137 (0.584-7.826) | 0.251 | 1.935 (0.438-8.551) | 0.384 |
| INR | 0.923 (0.809-1.053) | 0.232 | 0.954 (0.822-1.107) | 0.533 |
| FIB | 1.287 (0.997-1.661) | 0.053 | 1.157 (0.873-1.535) | 0.311 |
| **Notes:** adjusted sex, age, smoking, drinking, CAD, AF, baseline SBP, NIHSS on admission, MPV, PT, INR, FIB and leukocyte counts, anticoagulant therapy and antiplatelet therapy.  **Abbreviations:** CI, confidence interval; OR, odds ratio; HT, hemorrhagic transformation; HI, hemorrhagic infarct; PH, parenchymal hematoma; PLT, platelet counts; MPV, mean platelet volume; PT, prothrombin time; INR, International Normalized Ratio; FIB, fibrinogen. | | | | |
